# Structural Correlates of PPAR Agonist Rescue of Experimental Chronic Alcohol-Induced Steatohepatitis

**DOI:** 10.4172/2161-0681.1000114

**Published:** 2012-06-25

**Authors:** Teresa Ramirez, Ming Tong, Carol A. Ayala, Paul R. Monfils, Paul N. McMillan, Valerie Zabala, Jack R. Wands, Suzanne M. de la Monte

**Affiliations:** Liver Research Center and Departments of Medicine, Pathology, Neurology, and Neurosurgery, Rhode Island Hospital and The Warren Alpert Medical School of Brown University, Providence, RI, USA

**Keywords:** Alcoholic liver disease, Experimental model, PPAR agonists, Steatohepatitis, Insulin resistance, Histopathology, Electron microscopy, ER stress

## Abstract

Chronic alcoholic liver disease is associated with hepatic insulin resistance, inflammation, oxidative and ER stress, mitochondrial dysfunction, and DNA damage. Peroxisome-proliferator activated receptor (PPAR) agonists are insulin sensitizers that have anti-inflammatory/anti-oxidant effects. We previously showed that PPAR agonists can restore hepatic insulin responsiveness in chronic ethanol-fed rats with steatohepatitis. Herein, we furthered our investigations by characterizing the histological and ultrastructural changes mediated by PPAR agonist rescue of alcohol-induced steatohepatitis. Adult male Long Evans rats were pair fed with isocaloric liquid diets containing 0% or 37% ethanol (caloric) for 8 weeks. After 3 weeks on the diets, rats were treated with vehicle, or a PPAR-α, PPAR-δ, or PPAR-γ agonist twice weekly by i.p. injection. Ethanol-fed rats developed steatohepatitis with disordered hepatic chord architecture, mega-mitochondria, disruption of the RER, increased apoptosis, and increased 4-hydroxynonenal (HNE) and 3-nitrotyrosine (NTyr) immunoreactivity. PPAR-δ and PPAR-γ agonists reduced the severity of steatohepatitis, and restored the hepatic chord-like architectural, mitochondrial morphology, and RER organization, and the PPAR-δ agonist significantly reduced hepatic HNE. On the other hand, prominent RER tubule dilation, which could reflect ER stress, persisted in ethanol-exposed, PPAR-γ treated but not PPAR-δ treated livers. The PPAR-α agonist exacerbated both steatohepatitis and formation of mega-mitochondria, and it failed to restore RER architecture or lower biochemical indices of oxidative stress. In conclusion, improved hepatic insulin responsiveness and decreased inflammation resulting from PPAR-δ or PPAR-γ agonist treatments of alcohol-induced steatohepatitis are likely mediated by enhanced signaling through metabolic pathways with attendant reductions in ER stress, oxidative stress, and mitochondrial dysfunction.

## Introduction

World-wide, alcohol abuse is one of the leading causes of morbidity and mortality from chronic liver disease [1–3]. Chronic alcohol induced liver injury often progresses through stages from steatohepatitis to fibrosis, and then cirrhosis, culminating in liver failure or hepatocellular carcinoma [4,5]. Previous studies demonstrated that chronic Alcoholic Liver Disease (ALD) in humans and experimental animals was mediated by combined effects of hepatic insulin resistance [6–14] and hepatotoxic injury [15–18]. Alcohol-induced hepatic insulin resistance is caused by defects in intracellular signaling that occur at multiple levels within the cascade [19]. More specifically, ethanol inhibits hepatocellular insulin signaling by reducing insulin receptor binding, insulin receptor tyrosine phosphorylation, and activation of intrinsic receptor tyrosine kinase [6,7,20]. Ethanol also inhibits tyrosine phosphorylation of insulin receptor substrate proteins, which are major docking molecules used to transmit signals downstream from insulin and Insulin-Like Growth Factor (IGF) receptors [12,19]. Correspondingly, chronic ethanol exposure results in inhibition of Erk MAPK, which is needed for DNA synthesis and liver regeneration, and phosphatidylinositol-3-kinase (PI3 Kinase) [18], which promotes cell growth, survival, glucose utilization, and energy metabolism are impaired by chronic ethanol feeding [19,21]. Alcohol-induced hepatotoxicity is caused by inflammation, oxidative stress, mitochondrial dysfunction, and acetaldehyde-induced adduct formation [18]. The combined effects of chronic insulin/IGF resistance and hepatotoxicity cause sustained impairments in liver function mediated by constitutive inhibition of hepatocellular survival, energy metabolism, and DNA synthesis, and increased hepatocellular injury, DNA damage, oxidative stress, and activation of pro-apoptosis mechanisms [11,19,22,23]. Attendant inflammation and activation of stellate cells promote liver fibrosis and progression of alcoholic liver disease [24].

Peroxisome Proliferator-Activated Receptors (PPARs) are nuclear hormone receptors that bind to DNA and regulate gene transcription in a broad array of cell types and tissues [25–27]. PPARs are regulated by ligand binding, and they mediate their effects by heterodimerizing with the retinoid x receptor [25]. Three distinct isoforms of PPARs exist: PPAR-α, PPAR-δ (also referred to as PPAR-β), and PPAR-γ. PPAR-α is most abundantly expressed in brown adipose tissue and liver, followed by kidney, heart and skeletal muscle. PPAR-α is activated by polyunsaturated fatty acids and fibrates, and it regulates adipocyte growth and differentiation, lipid metabolism, lipoprotein synthesis, and tissue inflammatory responses [25,26,28]. PPAR-δ is widely expressed, but most abundant in gut, kidney and heart. PPAR-δ regulates expression of acyl-CoA synthetase 2 in brain, and it may participate in placental implantation and decidualization. In addition, PPAR-δ has a functional role in adaptive responses to the environment [26]. PPAR-γ is primarily expressed in adipose tissue, followed by colon, immune cells, and retina, whereas low levels are expressed in liver [25]. PPAR-γ influences storage of fatty acids in adipose tissue by regulating lipogenic metabolic and transport pathways [25,26,28]. Enhanced insulin sensitivity imparted by PPAR-agonist treatments led to their use in managing type 2 diabetes mellitus [28,29] and non-alcoholic fatty liver disease [28,30–32]. Moreover, PPAR-γ agonists were shown to prevent the development of alcohol-induced steatohepatitis [25,33].

In previous studies, we showed that PPAR-δ or PPAR-γ agonist treatments could restore several aspects of ethanol-impaired hepatic insulin/IGF resistance in a rat model of ALD [10,34]. For example, treatment with a PPAR-δ agonist normalized the regenerative capacity of the liver in chronic ethanol-exposed rats [10]. Moreover, treatment with a PPAR-δ or PPAR-γ agonist partly restored the liver architecture, enhance insulin/IGF responsiveness by increasing insulin/IGF receptor binding, gene expression, and signaling through phosphatidylinositol-3-kinase (PI3K), increasing hepatocyte gene expression, e.g. albumin, decreasing expression of pro-fibrogenic genes, e.g. collagen, and reducing indices of DNA damage, membrane lipid peroxidation, and oxidative stress [10,34].

Another important finding in these studies was that the different classes of PPAR agonists varied with respect to their restoration of liver function and hepatic architecture [34]. Since it appears that chronic ALD can be reversed or prevented by PPAR agonist treatments [34,35], it is imperative that we gain a better understanding of its mediators and identify additional attributes that correlate with corrective responses to therapy. The present study characterizes the nature and degree of PPAR agonist-induced structural and ultrastructural changes in livers of chronic ethanol exposed rats for correlation with previously established improvements in insulin/IGF responsiveness. Moreover, given the growing interest in the roles of lipotoxicity, endoplasmic reticulum (ER) stress, and mitochondrial dysfunction as mediators of steatohepatitis progression [19,21,36,37], it was of interest to determine if the therapeutic effects of PPAR agonists were associated with resolution of hepatic steatosis, ER morphology, and mitochondrial cytopathy.

## Methods

### Chronic ethanol exposure model

Adult male (~200–250 g) Long Evans rats (Harlan Sprague Dawley, Inc., Indianapolis, IN) were pair-fed with isocaloric liquid diets (BioServ, Frenchtown, NJ) containing 0% (control) or 37% ethanol by caloric content (9.2% v/v) for 8 weeks [7,34]. Rats were monitored daily to ensure equivalent food consumption and maintenance of body weight. After 3 weeks of liquid diet feeding, the rats were administered twice weekly (Mondays and Thursdays) intra-peritoneal (i.p.) injections of vehicle (saline), a PPAR-α (GW7647; 25 μg/Kg), PPAR-δ (L-160,043; 2 μg/Kg), or PPAR-γ (F-L-Leu; 20 μg/Kg) agonist (CalBiochem, Carlsbad, CA) for 6 weeks (N=12/group). The PPAR doses, routes of administration, and frequency were based on previous experimental protocols [10,34]. At the conclusion of the experiment, rats were anesthetized with vaporized isofluorane (Novaplus, Irving, TX), and livers were harvested for study. Liver samples were fixed for histological and ultrastructural studies (see below), and snap-frozen and stored at −80°C for mRNA and protein studies. Rats were housed under humane conditions and kept on a 12-hour light/dark cycle with free access to food. The protocols for using vertebrate animals for this research conformed to guidelines established by the National Institutes of Health and were approved by the Institutional Animal Care and Use Committee at the Lifespan-Rhode Island Hospital.

### Histological studies

Liver tissue was fixed in 10 volumes of 10% buffered paraformaldehyde and embedded in paraffin. Histological sections (5 μm) were stained with Hematoxylin and Eosin (H&E) (Surgipath Medical Industries, Inc., Richmond, IL), Sirius Red (Rowley Biochemical, Inc., Danvers, MA), or Periodic Acid-Schiff (PAS) reagent (Fisher Scientific, Houston, TX and Surgipath Medical Industries, Inc., Richmond, IL), for routine histological studies, and to detect fibrosis and glycogen deposition, respectively. In addition, paraformaldehyde fixed liver tissue was embedded in Optimum Cutting Temperature (O. C. T.) compound (Sakura Finetek, Inc., Torrence, CA), and cryostat sections (10 μm thick) were stained with Oil Red O (Rowley Biochemical, Inc., Danvers, MA) to detect neutral lipids. Oil Red O stained sections were counterstained lightly with Harris’s hematoxylin.

### Electron Microscopy (EM)

Liver tissue cut into 1 mm cubes, was immersion fixed over night at 4°C in standard EM fixative containing 4% paraformaldehyde and 2.5% glutaraldehyde in 0.15M sodium cacodylate buffer, pH 7.4. Subsequently, the tissue was rinsed in cacodylate buffer and post-fixed in 1% osmium tetroxide. Following post-fixation rinses in cacodylate buffer and dehydration through a graded acetone series, the samples were embedded in Spurr’s epoxy resin (Ladd Research, Williston, VT) and the blocks were allowed to polymerize over night at 70°C. Semi-thin sections (1μ) generated with a Reichert Ultracut S microtome, were stained with toluidine blue. Ultra-thin sections (50–60 nm) placed onto 300 mesh copper grids (Electron Microscopy Sciences, Hartfield, PA), were contrasted with uranyl acetate and lead citrate, and examined with a Morgagni 268 transmission electron microscope. Images were collected with an AMT Advantage 542 CCD camera system.

### Enzyme-linked immunosorbant assay (ELISA)

Protein expression was assessed by ELISA and liver lipid content was measured using the Nile Red fluorometric assay as previously described [34], 22. Liver tissue samples were homogenized in 5-volumes of radioimmunoprecipitation assay (RIPA) buffer containing protease and phosphatase inhibitors [7,34]. Protein concentrations were determined using the bicinchoninic acid (BCA) assay (Pierce, Rockford, IL). Protein homogenates diluted in TBS (40 ng/100 μl) were adsorbed to the bottoms of the wells by overnight incubation at 4°C. Non-specific binding sites were blocked with 2% bovine serum albumin (BSA) in TBS. Samples were incubated with primary antibody (0.01–0.1 μg/ml) for 1 hour at room temperature. Immunoreactivity was detected with HRP-conjugated secondary antibody (1:10000; Pierce) and the Amplex Red soluble fluorophore (Invitrogen, Grand Island, NY). Amplex Red fluorescence was measured (Ex 530/Em 595) in an M-5 machine (fluorescence light units; FLU). Negative controls included incubations with non-relevant antibodies, or with the primary or secondary antibody omitted. The mean levels of specific immunoreactivity were used for inter-group statistical comparisons.

### Statistical Analysis

Data depicted in box plots reflect the median (horizontal bar), 95% confidence interval limits (upper and lower boundaries of boxes), and range (whiskers). Inter-group comparisons were made using two-way analysis of variance (ANOVA) with the Bonferroni post-hoc test for significance. Statistical analyses were performed using GraphPad Prism 5 software (GraphPad Software, Inc., San Diego, CA). Significant P-values (P<0.05 or better) are indicated within the graph panels.

## Results

### Histopathological effects of PPAR agonist treatments on chronic ethanol-induced steatohepatitis

**Figure 1:**
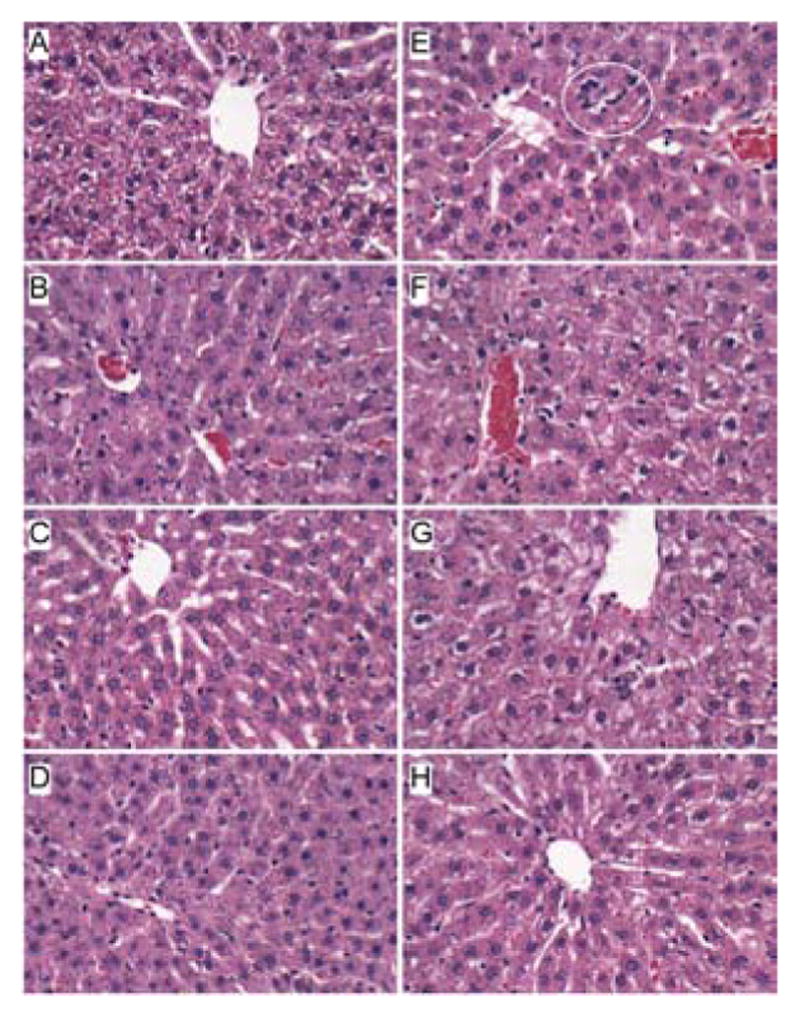
Histologic effects of PPAR agonists on alcohol-induced steatohepatitis. Adult male Long Evans rats were chronically fed with liquid diets containing (A–D) 0% (control) or (EH) 37% ethanol, and treated by i.p. injection of (A,E) vehicle (saline), or a (B,F) PPAR-α (GW7647; 25 μg/Kg), (C,G) PPAR-δ (L-160,043; 2 μg/Kg), or (D,H) PPAR-γ (F–L-Leu; 20 μg/Kg) agonist (See Materials and Methods). Formalin-fixed paraffin-embedded sections (5 μm thick) of liver were stained with H&E. Zone 3 hepatocytes are depicted at or near the center of each image. Note regular chord-like architecture, uniform hepatocyte morphology and absence of steatosis in control livers, the loss of chord-like architecture, steatosis (arrow) and inflammatory infiltrate (circle) in the ethanol-exposed, vehicle-and PPAR-α agonist treated livers, and restoration of hepatic architecture in ethanol+ PPAR-δ and PPAR-γ treated livers. Original magnifications, 200x.

**Figure 2:**
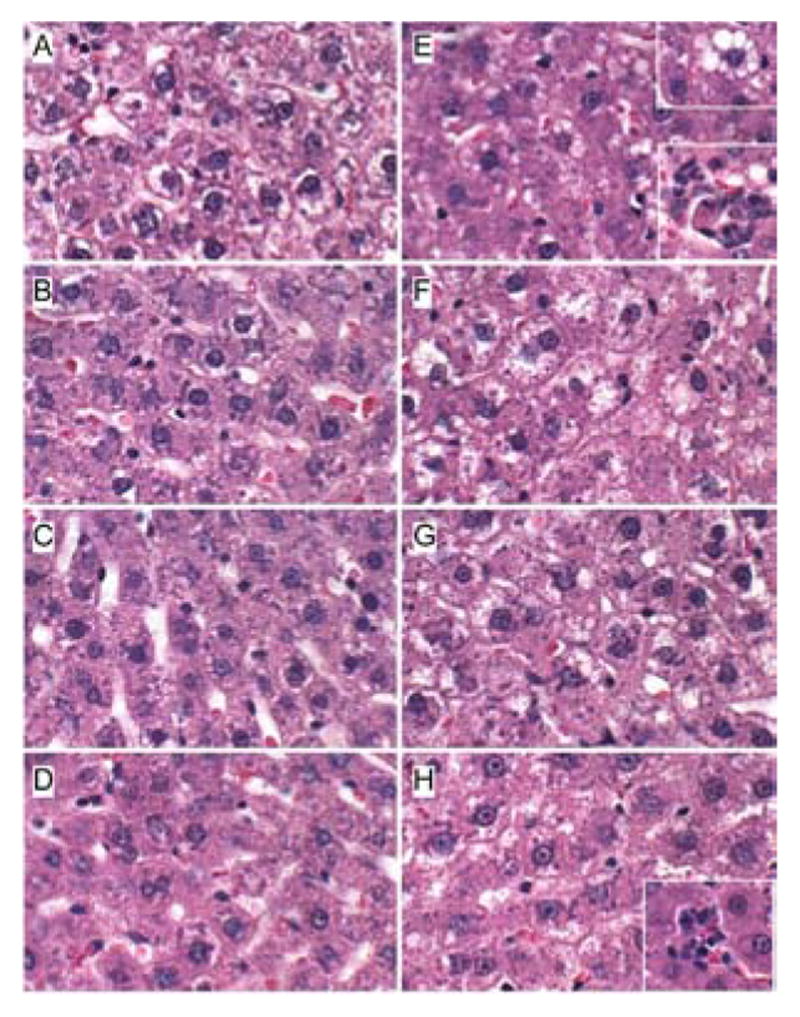
Histologic responses to PPAR agonist treatments of experimental chronic alcohol-induced steatohepatitis. Adult male Long Evans rats were fed with diets containing (AD) 0% or (E–H) 37% ethanol and treated with (A,E) vehicle, or a (B,F) PPAR-α, (C,G) PPAR-δ, or (D,H) PPAR-γ agonist. Formalinfixed paraffin-embedded sections (5 μm thick) of liver were stained with H&E. Zone 3 hepatocytes are depicted. Note the uniform hepatocyte cytology in control livers versus the prominent steatosis (clear cytoplasmic vacuoles, e.g. upper right inset in E), inflammation (lower insets in E and H) and apoptosis (lower inset in E) in the ethanol-exposed, vehicle-, PPAR-α, or PPAR-γ treated livers. Original magnifications, 400x.

H&E stained sections demonstrated that control livers, irrespective of PPAR agonist treatment, exhibited the expected well-organized lobular architecture with hepatocytes arranged in chords with regularly aligned sinusoids. The hepatocytes were relatively uniform in size, and the livers had little or no evidence of chronic inflammation, necrosis, steatosis, or apoptosis (Figures 1A–1D). At 400x magnification, control liver hepatocytes had variable degrees of cytoplasmic clearing (Figure 2A), which corresponded to glycogen accumulation, as demonstrated by PAS staining (data not shown). PPAR agonist treatments reduced the cytoplasmic clearing (Figures 2B–2D) and PAS staining intensity, and rendered the cytoplasm homogeneously amphophilic or eosinophilic (Figures 2B–2D).

Livers of chronic ethanol fed, vehicle-treated rats had prominent microvesicular and macrovesicular steatosis, multiple foci of intralobular lympho-mononuclear cell inflammation, scattered apoptotic cells, micro-foci of necrosis, increased variability in hepatocyte nuclear size, and lobular disarray with loss of the regular chord-like architecture (Figures 1E and 2E). Treatment with the PPAR-α agonist did not improve the overall histology of ethanol-exposed livers; however, it did convert the mainly macrovesicular steatosis to microvesicular steatosis (Figures 1F and 2F). In contrast, treatment with either the PPAR-δ (Figures 1G and 2G) or PPAR-γ (Figures 1H and 2H) agonist reduced the steatosis, apoptosis, and variability in hepatocyte size, and nearly restored the normal chord-like architecture. In addition, the PPAR-γ agonist treatments reduced intra-lobular inflammation, whereas the PPAR-δ agonist treatments did not (Figures 2G–2H).

### Further analysis of PPAR agonist effects on hepatic steatosis

**Figure 3:**
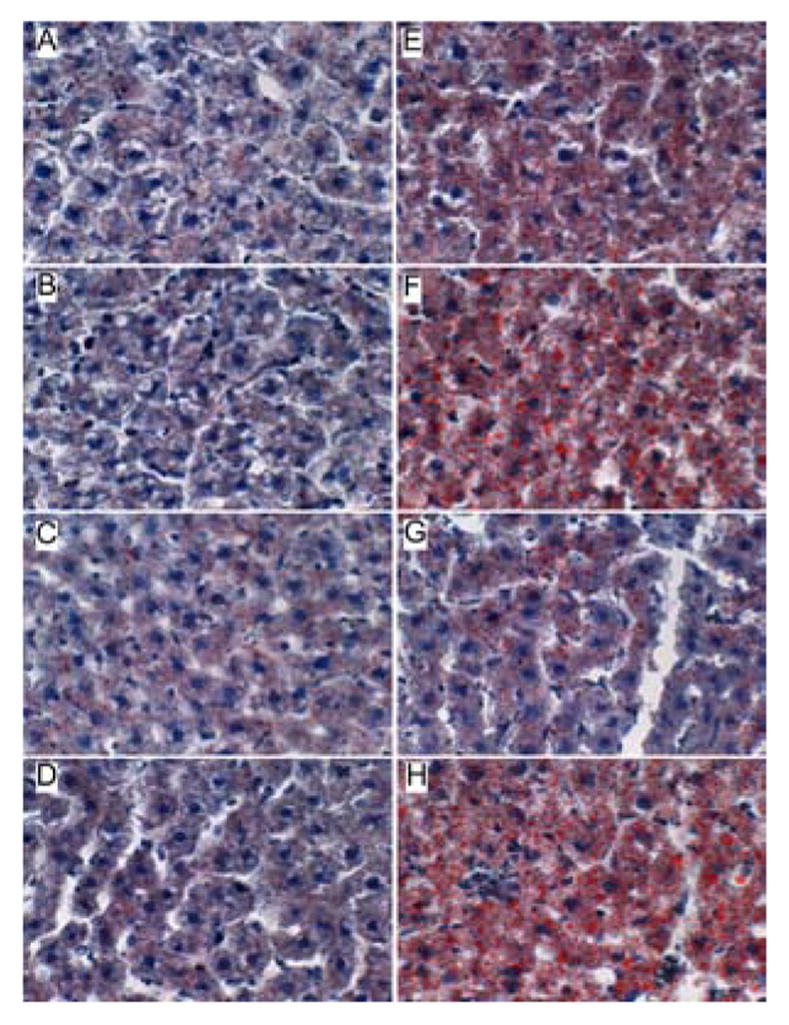
Effects of PPAR agonists on ethanol-induced hepatic steatosis. Formalinfixed, cryostat sections (10 μm thick) of liver from (A–D) control or (E–H) ethanol-fed rats treated with (A,E) vehicle, or a (B,F) PPAR-α, (C,G) PPAR-δ, or (D,H) PPAR-γ agonist were stained with Oil Red O to detect cytoplasmic lipid accumulation (red punctate labeling). Note strikingly lower levels of Oil Red O staining in (A–D) control and (H) ethanol+PPAR-δ agonist treated livers compared with (E,F,H) ethanol-fed and vehicle, PPAR-α, or PPAR-γ agonist treated livers. Original magnifications, 400x.

Oil Red O staining demonstrated low levels of microvesicular cytoplasmic lipid accumulation in hepatocytes mainly distributed in Zone 3 (perivenous) (Figure 3A). In contrast, Zones 1 (periportal) and 2 (midzonal) hepatoacytes were virtually negative for Oil Red O staining. Although the PPAR agonist treatments altered the levels of baseline hepatic steatosis, Oil red O staining was mainly distributed in Zone 3 hepatocytes. PPAR-α agonist treatment of control rats increased the intensity of Oil red O staining (Figure 3B), whereas treatments with the PPAR-δ agonist strikingly reduced Oil red O staining, and therefore hepatic steatosis (Figure 3C). Livers from ethanol-fed, PPAR-γ agonist treated rats had similar levels and distributions of hepatic steatosis relative to corresponding vehicle-treated controls (Figure 3D).

Chronic ethanol feeding resulted in strikingly higher levels of hepatocellular Oil Red O staining relative to vehicle-treated controls. Ethanol+vehicle exposed livers had pan-lobular steatosis such that hepatocytes throughout the lobules and in all three zones contained abundant microvesicular and macrovesicular cytoplasmic lipid droplets (Figure 3E). Treatment with the PPAR-α agonist further increased hepatic steatosis in the ethanol-fed group, although the steatosis was mainly macrovesicular in nature (Figure 3F). In rats treated with the PPAR-δ agonist, steatosis was sharply reduced such that the Oil red O staining was more limited to Zone 3 and low-level in intensity, similar to the findings in the control+vehicle group (Figure 3G). Finally, PPAR-γ agonist treatment of chronic ethanol-fed rats had similar levels of Oil red O staining as noted for the corresponding vehicle-treated group (Figure 3H). The higher lipid content in ethanol-fed versus control livers, and the histological effects of the PPAR agonists detected in histological sections were reflected by similar trends measured by the Nile Red assay (Table 1).

### Effects of PPAR agonist treatments on hepatic fibrosis

**Figure 4:**
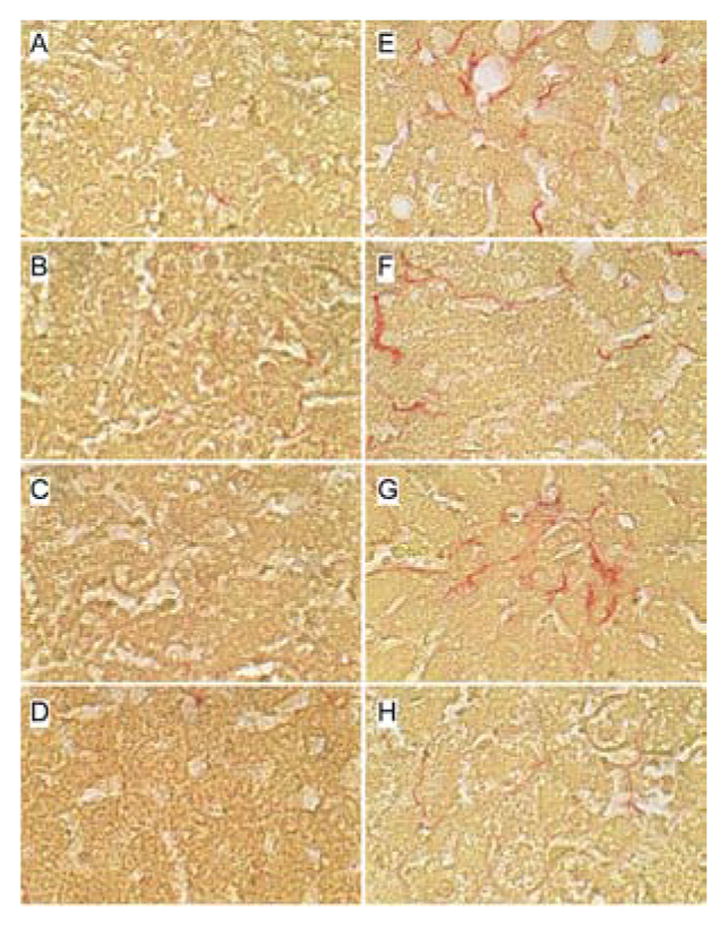
Effects of PPAR agonists on ethanol-induced hepatic fibrosis. Formalin-fixed, paraffin-embedded sections (5 μm thick) of liver from (A–D) control or (E–H) ethanol-fed rats that were treated with (A,E) vehicle, or a (B,F) PPAR-α, (C,G) PPAR-δ, or (D,H) PPAR-γ agonist were stained with Sirius red to detect collagen (wavy red linear labeling). Note minimal fibrosis in (A-D) control and (H) ethanol+PPAR-γ agonist treated livers compared with the (EG) delicate peri-hepatocyte and sinusoidal labeling of collagen fibrils in ethanol-exposed vehicle, PPAR-α, or PPAR-δ agonist treated livers. Original magnifications, 400x.

Sirius Red stained sections of liver revealed minimal collagen fibril deposition in control rats, irrespective of PPAR agonist treatment (Figures 4A–4D). Chronic ethanol feeding (+vehicle treatment) resulted in conspicuously increased levels and distribution of coarse and fine peri-hepatocyte and sinusoidal collagen deposits. In addition, there was focal “chickenwire fibrosis” characterized by a trabecular or mesh-like pattern of collagen fibrils that completely surrounded individual hepatocytes (Figure 4E). Treatment with either the PPAR-α or PPAR-δ agonist slightly altered the character and degree of Sirius red staining associated with chronic ethanol feeding. In the PPAR-α-treated group, the collagen fibrils were mainly coarse and linear, and tracked along the sinusoids (Figure 4F), whereas in the PPAR-δ-treated group, the fibrosis was fine and mesh-like rather than linear (sinusoidal) (Figure 4G). The treatment with PPAR-γ agonist nearly abrogated the fibrosis (Figure 4H). See summarized results in (Table 2).

### Fine histological features of chronic alcohol-induced steatohepatitis and effects of PPAR agonist treatment

**Figure 5:**
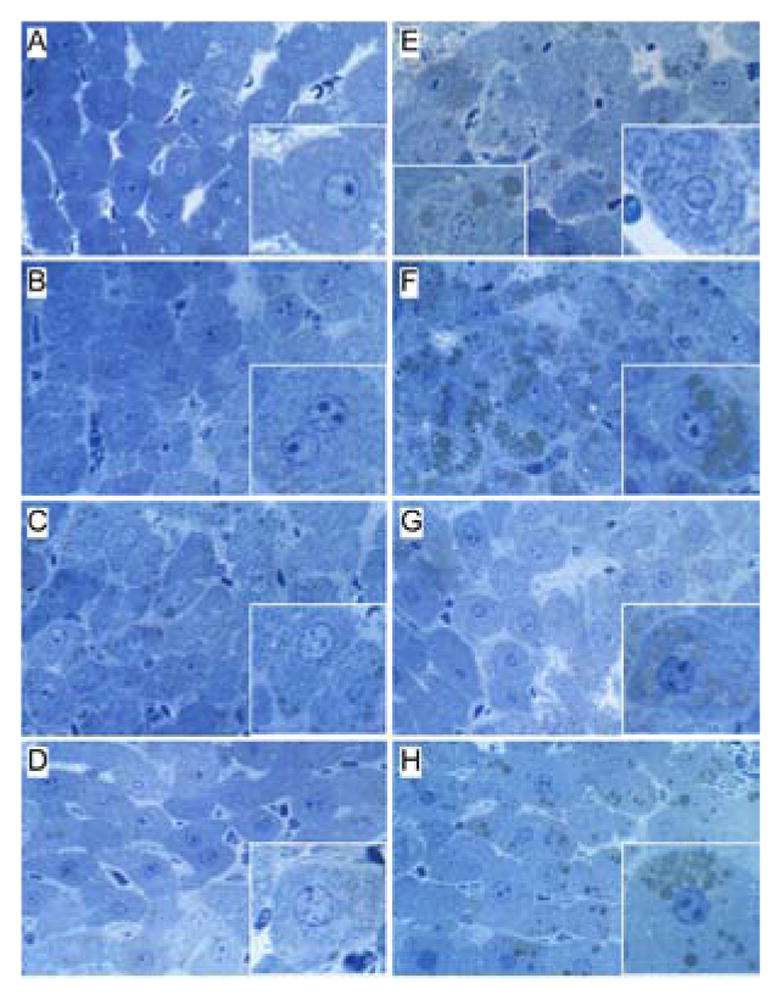
Fine histological features of chronic alcohol-induced liver disease and the effects of PPAR agonist treatments. Liver tissue from adult male rats that were chronically fed with (A–D) 0% or (E–H) 37% ethanol-containing liquid diets, and treated with (A,E) vehicle, or a (B,F) PPAR-α, (C,G) PPAR-δ, or (D,H) PPAR-γ agonist was embedded in Spurr’s epoxy resin. 1 μm thick sections were stained with Toluidine blue. Note uniform cytoplasm with minimal lipid droplets in (A–D) control and (G) ethanol+PPAR-δ agonist treated livers, and (E,F,H) prominent lipid droplets (greenish-gray, insets) and (E) mega-mitochondria (coarse cytoplasmic granularity) in ethanol-exposed livers. Original magnifications-400x; insets-1200x.

**Figure 6:**
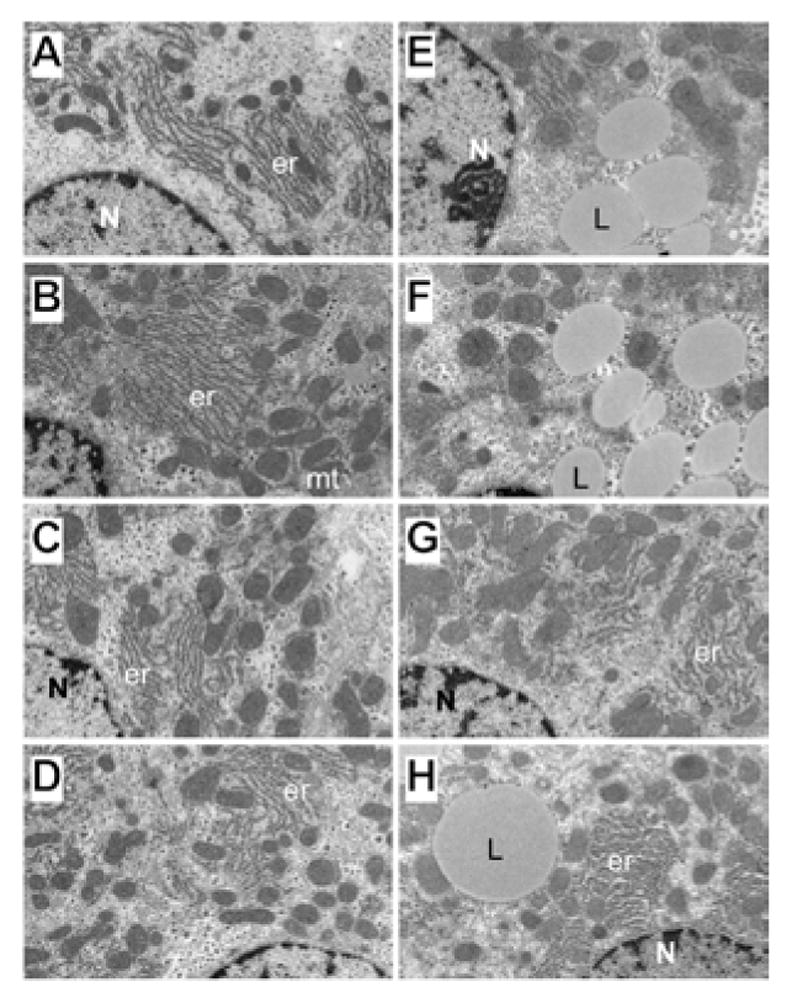
Ultrastructural features of alcohol-induced liver injury and PPAR agonist effects. Adult male Long Evans rats were chronically fed with liquid diets containing (A–D) 0% or (E–H) 37% ethanol, and administered i.p. injections of (A,E) vehicle, or a (B,F) PPAR-α, (C,G) PPAR-δ, or (D,H) PPAR-γ agonist. Spurr’s resin-embedded, 50–60 nm thick sections of liver were contrasted with uranyl acetate and lead citrate and examined by EM. (A–D, G, H) Note the regular parallel RER stacks (er) with mitochondria (mt) distributed mainly along the periphery but in close proximity to the RER. (E,F,H) Chronic ethanol feeding resulted in increased density of lipid droplets (L), irregular shape and enlargement of mitochondria, and disruption of the RER architecture. N=nucleus. (Original magnifications, 7100x).

Semi-thin 1 μM-thick sections of liver revealed relatively uniform hepatocyte cytology in control samples, irrespective of PPAR agonist treatments (Figures 5A–5D). However, subtle changes, including increased abundance of cytoplasmic vacuoles in PPAR-α-treated livers, and prominent dense oval to round punctate bodies corresponding to mitochondria in PPAR-δ-treated livers were noted in hepatocytes (Figures 5A–5C). Hepatocytes in ethanol-exposed livers varied in size, had less prominent nucleoli, and contained abundant micro-vesicular as well as macrovesicular cytoplasmic lipid droplets (Figure 5E). Corresponding with the Oil red O staining results, PPAR-α agonist treatment of ethanol-exposed livers further increased the levels of hepatic steatosis and resulted in cytoplasmic accumulations of mainly macro-vesicular lipid droplets (Figure 5F). Treatment with the PPAR-δ agonist strikingly reduced the levels of hepatocellular steatosis and variability in cell size (Figure 5G). PPAR-γ agonist treatment of chronic ethanol-exposed livers had both micro- and macrovesicular steatosis that was more clustered rather than diffusely distributed in the cytoplasm (Figure 5H). In addition, both PPAR-δ and PPAR-γ agonist treatments resulted in increased prominence of hepatocyte nucleoli. Ultrastructural features of chronic alcohol-induced steatohepatitis and effects of PPAR agonist treatment: Transmission EM studies were focused on characterizing the structure and spatial distribution of the ER and mitochondria. Relatively low magnification studies (7100x) demonstrated that in control+vehicle treated livers, the RER cisternae (flattened/narrowed, elongated tubules) were stacked in parallel arrays and closely juxtaposed to mitochondria that were mainly localized along the periphery of the ER stacks (Figure 6A). Mitochondria were relatively uniform in size and oval to elongate in shape. Glycogen granules were diffusely distributed and lipid droplets were scarce. PPAR-α agonist treatment increased the compactness of the RER, size of mitochondria and glycogen granules, and density of lipid droplets. The spatial relationship between RER and mitochondria was similar to that observed in control+vehicle treated samples (Figure 6B). PPAR-δ and PPAR-γ agonist treatments resulted in less compact organization of the RER and distribution of mitochondria among the RER rather than along the periphery (Figures 6C and 6D). In addition, the RER in the PPAR-γ treated livers was somewhat dispersed and disorganized (Figure 6D). Mitochondria were enlarged in PPAR-δ treated livers and more varied in size in the PPAR-γ treated livers. Glycogen granules were prominently enlarged in both PPAR-δ and PPAR-γ treated livers, similar to the findings with respect to PPAR-α treatment.

Chronic ethanol feeding with vehicle treatment resulted in increased densities of lipid droplets and glycogen granules, mitochondrial pleomorphism with increased variability in mitochondrial size as well as irregular and striking enlargement of mitochondria (megamitochondria), and disorganization and dispersal of the RER. Mitochondrial cristae and matrix were relatively preserved. However, with regard to the RER, instead of being uniformly arranged in parallel stacks, multiple regions of RER exhibited subtotal loss of the normal architecture. In areas where narrowed tubular RER structures were detected, the ribosomes were irregularly spaced and cisternae were irregularly dilated. Mitochondria were distributed amongst the RER rather than at the perimeters of stacked clusters (Figure 6E). PPAR-α treatment of ethanol-fed rats increased the density of lipid droplets and the size of glycogen granules, but did not result in better organization of the RER (Figure 6F). In contrast, PPAR-δ and PPAR-γ agonist treatments reduced the density of lipid droplets and increased the organization and compactness of RER. In addition, the mitochondria were rendered smaller, more uniform, and peripherally distributed relative to the RER stacks (Figures 6G–6H).

**Figure 7:**
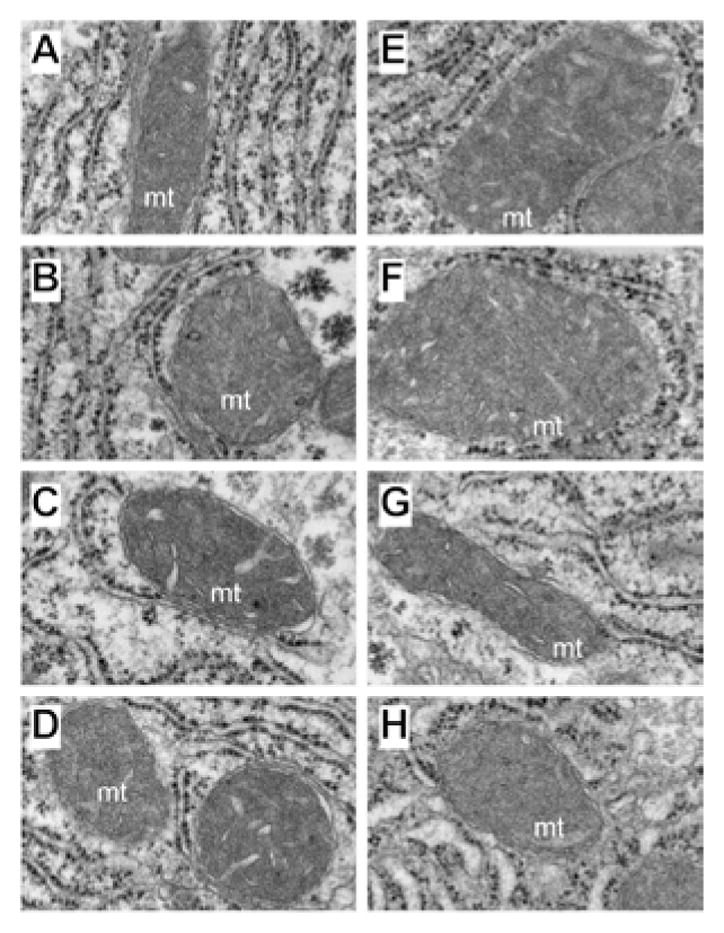
Chronic ethanol feeding and PPAR agonist treatment effects on mitochondrial structure. Adult male Long Evans rats were fed with liquid diets containing (A–D) 0% or (E–H) 37% ethanol for 8 weeks, and administered i.p. injections of (A,E) vehicle, or a (B,F) PPAR-α, (C,G) PPAR-δ, or (D,H) PPAR-γ agonist. Spurr’s resin-embedded, 50–60 nm thick sections of liver were contrasted with uranyl acetate and lead citrate and examined by EM. (A–D) Control livers had relatively uniform size and shape of mitochondria with complex matrix/cristae. (E,F) Ethanol+vehicle and ethanol+PPAR-α agonist treated livers exhibited mega-mitochondria that displaced and distorted the RER. (G,H) Ethanol+PPAR-δ and ethanol+PPAR-γ agonist treated livers had mitochondria that were similar to control. Original magnifications, 44000x.

Higher magnification studies (44000x) demonstrated complex mitochondrial cristae in all control groups, but moderate enlargement of mitochondria with distortion/displacement of the RER in PPAR-agonist treated relative to control+vehicle treated livers (Figures 7A–7D). In addition, large glycogen aggregates were readily detected in the cytoplasm of PPAR-α (Figure 7C) and PPAR-δ (not shown) exposed hepatocytes. Chronic ethanol feeding + vehicle treatment resulted in prominent enlargement of mitochondria (giant mitochondria) that physically displaced/distorted the RER tubules (Figure 7E). PPAR-α treatment resulted in further enlargement of mitochondria and distortion of the RER (Figure 7F). In the ethanol + PPAR-δ agonist treated livers, mitochondrial morphology and size were nearly restored to normal and were similar to those seen in control+vehicle treated livers, although RER distortion including focal tubular dilation persisted (Figure 7G). PPAR-γ agonist treatment of chronic ethanol-fed rats was also associated with restoration of the normal mitochondrial size although the RER tubules were still distorted by the inter-position of mitochondria (Figure 7H).

**Figure 8:**
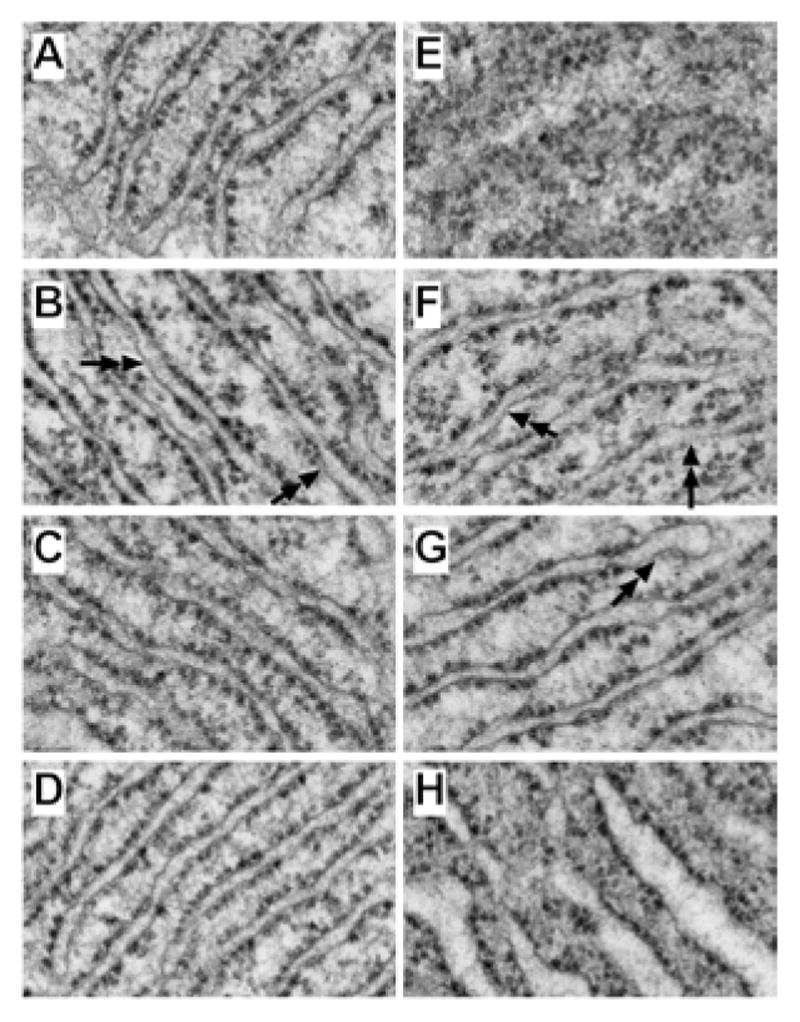
Chronic ethanol exposure and PPAR agonist treatment effects on RER structure. Adult male Long Evans rats were fed with liquid diets containing (A–D) 0% or (E–H) 37% ethanol for 8 weeks, and administered i.p. injections of (A,E) vehicle, or a (B,F) PPAR-α, (C,G) PPAR-δ, or (D,H) PPAR-γ agonist. Spurr’s resin-embedded, 50–60 nm thick sections of liver sections were contrasted with uranyl acetate and lead citrate and examined by EM. (AD) Control livers had relatively uniform parallel stacks of flattened RER cisternae with regularly spaced ribosomes. PPAR-α agonist treatments resulted in patchy absence of ribosomes (arrowheads) on the ER in both (B) control and (F) ethanol-exposed livers. (E) Ethanol+vehicle treated livers had disrupted, disorganized RER. This phenomenon was prevented by (F–H) PPAR agonist treatment. However, in ethanol-exposed livers, the RER cisternae were variably dilated and irregularly populated by ribosomes. Original magnification, 71000x.

The highest magnification studies (71000x) were used to characterize the effects of ethanol and PPAR agonist treatments on RER structure (Figure 8). In control+vehicle samples, the RER tubules were relatively uniform and parallel with somewhat even and regular spacing of ribosomes on the cytoplasmic surfaces (Figure 8A). PPAR-α agonist treatment resulted in increased variability in RER luminal diameters and patchy loss of ribosomes, rendering the surfaces smooth (Figure 8B). The RER of PPAR-δ and PPAR-γ agonist treated livers were highly regular and somewhat more densely populated with ribosomes relative to control+vehicle treated livers (Figures 8C–8D). Chronic ethanol feeding plus vehicle treatment resulted in regionally severe disorganization of the ER with loss of the tubular and secular architecture, and irregular aggregation of ribosomes (Figure 8E). PPAR agonist treatments restored the RER tubular architecture to variable degrees. PPAR-α and PPAR-δ agonist treatments produced the best effects in terms of rendering the RER tubules more uniform, parallel, and well populated by ribosomes on the outer surfaces, although free ribosome aggregates and areas of ribosome-free ER were readily detected in the ethanol+PPAR-α agonist treated samples (Figure 8F–8G). Treatment of chronic ethanol-exposed livers with the PPAR-γ agonist was associated striking dilatation of ER tubules, although the cytoplasmic surfaces were well populated with ribosomes (Figure 8H).

### Effects of PPAR agonist treatments on pro-inflammatory cytokine and oxidative stress markers

**Figure 9:**
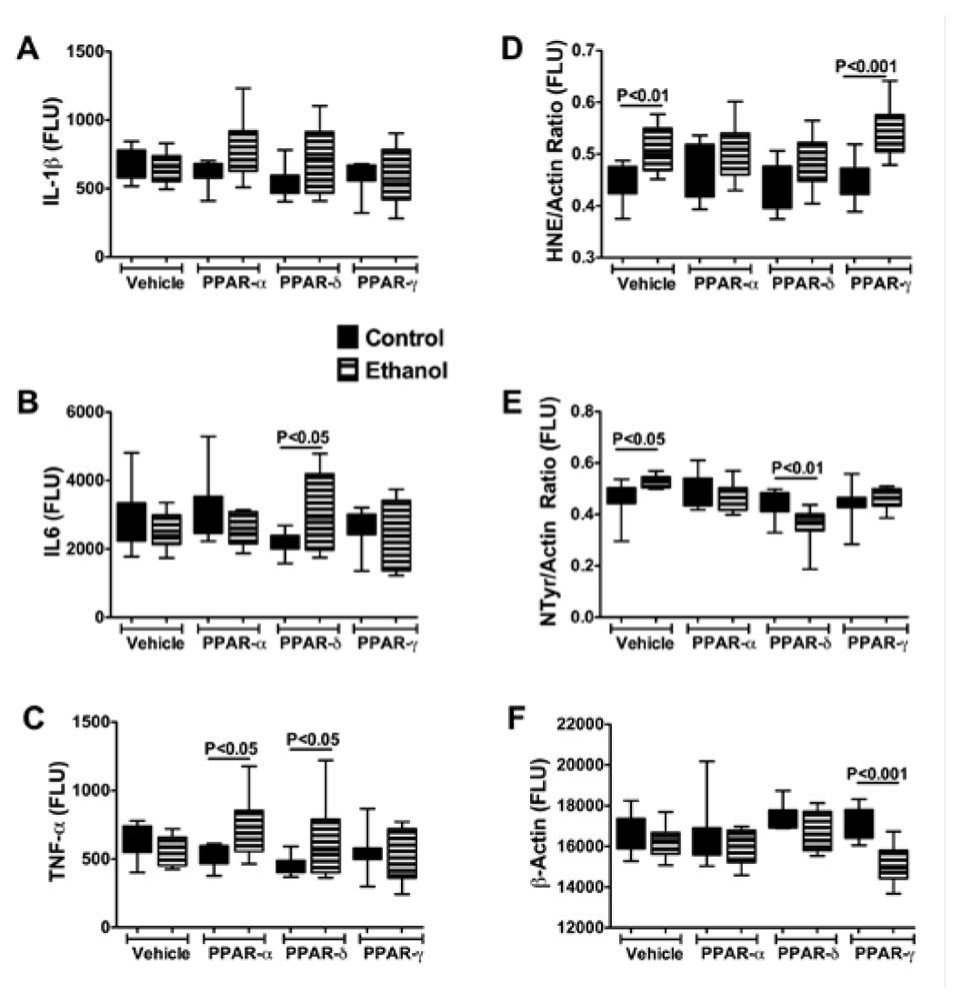
Effects of chronic ethanol feeding and PPAR agonist treatments on inflammatory cytokine and oxidative stress indices in liver. (A) IL-1β, (B) IL6, (C) TNF-α, (D) 4-hydroxynonenal (HNE), (E) 3-nitrotyrosine (NTyr), and (F) β-actin were measured in liver homogenates by (A–C) multiplex or (D–F) duplex ELISAs. Inter-group comparisons were made using two-way ANOVAs with Bonferroni posttests (N=12 samples/group). P-values corresponding to significant within-treatment group differences (P<0.05 or better) are indicated.

ELISAs were used to measure immunoreactivity corresponding to Interleukin-1β (IL-1β), IL-6, tumor necrosis factor-alpha (TNF-α), 4-hydroxy-2-nonenal (4-HNE), 3-nitrotyrosine (N-Tyr), and β-Actin. IL-1β, IL-6, and TNF-α were measured because these cytokines have demonstrated roles as mediators of injury in both alcoholic and nonalcoholic steatohepatitis [5,38–40], and PPAR agonists function in part by regulating proinflammatory responses [28]. 4–HNE, a marker of lipid peroxidation in ALD, and 3-N-Tyr, which marks peroxynitrite accumulation, reflect adduct accumulation and oxidative stress in ALD [16,41–43]. The results, including 12 samples per group, were analyzed using two-way ANOVA tests with Bonferroni posttests. A significant overall difference between the control and ethanol exposed groups was observed with respect to IL-1β (F=4.192; P=0.04) with higher levels detected in the ethanol+PPAR-α and ethanol+PPAR-δ relative to corresponding controls. However, posthoc tests did not reach statistical significance (Figure 9A). With respect to both IL-6 (F=12.54; P=0.007) and TNF-α (F=14.29; P=0.0018), significant intergroup differences corresponding to interactions between ethanol exposure and PPAR agonist treatments were detected, and the Bonferroni posthoc test demonstrated significantly higher levels of IL-6 in the ethanol+PPAR-δ (P<0.05) and TNF-α in the ethanol+PPAR-α (P<0.05) and ethanol+PPAR-δ (P<0.05) groups relative to corresponding controls (Figures 9B–9C).

Significant inter-group differences were observed with respect to 4-HNE, N-Tyr, and β-Actin. The two-way ANOVA test detected highly significant inter-group differences with respect to 4-HNE (F=27.34; P<0.0001), and the posthoc Bonferroni test demonstrated significantly higher mean levels of 4-HNE in the ethanol+vehicle (P<0.01) and ethanol+PPAR-γ agonist (P<0.001) treated relative to corresponding controls. In contrast, the mean levels of 4-HNE were similar in control and ethanol-exposed groups that were treated with either the PPAR-α or PPAR-δ agonist (Figure 9D).

Significant within group effects of PPAR agonist treatments (F=24.07, P<0.0001) and interactions between ethanol feeding and PPAR agonist treatments (F=15.53, P=0.001) were observed with respect to N-Tyr immunoreactivity in the livers. The mean levels of N-Tyr immunoreactivity were similar among all control sub-groups except PPAR-α, which had the highest level. Among the ethanol-exposed subgroups, hepatic N-Tyr immunoreactivity was highest in the vehicle-treated group and lowest in the PPAR-δ agonist treated group. Posthoc tests demonstrated significantly higher levels of N-Tyr in the ethanol+vehicle treated (P<0.05), and lower levels in the ethanol+PPAR-δ agonist treated (P<0.01) relative to corresponding controls. Therefore, chronic ethanol exposure-induced oxidative stress responses manifested by increased HNE and N-Tyr immunoreactivity in the liver, and PPAR-δ agonist treatments abrogated these effects.

β-actin expression was consistently lower in ethanol-exposed relative to control livers (F=23.28; P<0.0001). Posthoc testing demonstrated that the mean level of β-actin was significantly lower in the ethanol+PPAR-γ agonist treated relative to the corresponding control group (P<0.001) (Figure 9F). Moreover, β-actin immunoreactivity was lowest in the ethanol+PPAR-γ agonist treated relative to all other groups, both control and ethanol exposed (F=11.28, P=0.0026). Although ethanol is known to induce actin cytoskeletal disorganization and disruption of tight junctions in liver [44], it is unclear why the PPAR-γ agonist treatments produced such striking effects on β-actin protein expression, i.e. probable degradation.

## Discussion

This study provides the first detailed analysis of the histological and ultrastructural effects of insulin sensitizer treatments on chronic alcohol-induced steatohepatitis. The findings shed light on the mechanisms by which chronic ethanol abuse impairs liver function, and how the different classes of PPAR agonists mediate their therapeutic effects in liver. Corresponding with previous reports, chronic ethanol feeding in the Long Evans rat model produces significant steatohepatitis with persistent inflammation, hepatocellular apoptosis and necrosis, and increased cell turnover with poor remodeling which results in disorganization of the lobular architecture. The progressive nature of the steatohepatitis is marked by the increased chicken-wire fibrosis detected by Sirius Red staining. Ethanol-induced increases in hepatic neutral lipid as demonstrated with the Nile red assay were associated with pan-lobular steatosis as shown by Oil red O staining. Examination of 1-micron thick sections by light microscopy and ultrathin sections by transmission EM revealed that chronic ethanol feeding resulted in mitochondrial pleomorphism with mega-mitochondria, abundant accumulations of variable size cytoplasmic lipid droplets, and prominent disorganization and disruption of RER tubules. These findings are quite reminiscent of previously reported ultrastructural changes associated with alcohol-induced liver disease in humans [45–50].

Light microscopic and ultrastructural studies demonstrated that PPAR agonist treatments abrogate some of the adverse effects of chronic ethanol feeding on hepatic structure, although the nature and degree of responses varied among the different classes of PPAR agonists. The PPAR-α agonist treatments either worsened or had no detectable impact on the histological and ultrastructural pathology in ethanol-exposed livers, as was manifested by the increased lipid storage and similar degrees of inflammation, fibrosis, megamitochondria, and RER disruption. In contrast, the PPAR-δ and PPAR-γ agonists had substantially better therapeutic effects in that they both reduced inflammation, steatosis, mega-mitochondria, and RER disruption, and restored the hepatic lobular architecture. Moreover, treatment with the PPAR-γ agonist reduced lobular fibrosis as demonstrated by Sirius Red staining. It is noteworthy that our previous studies demonstrated that PPAR-δ and PPAR-γ agonists were more effective than PPAR-α agonists in restoring hepatic insulin and IGF responsiveness and gene expression in chronic ethanol-exposed livers [34]. Therefore, the present work links PPAR agonist-mediated restoration of liver structure with function in the context of continued high-level ethanol consumption. The results suggest that by supporting insulin/IGF signaling networks with the appropriate classes of PPAR agonists, the severity of hepatic steatosis and abundance of mega-mitochondria decline, and the cascade leading to liver degeneration, including ER stress (manifested by disruption of the tubular architecture), mitochondrial dysfunction (mega-mitochondria), and fibrogenesis (increased Sirius red staining of sinusoidal and peri-hepatocyte collagen) can be curtailed or interrupted. The findings also support the hypothesis that impairments in insulin/IGF signaling in the liver drive progression of ethanol-induced steatohepatitis.

Further studies examined the effects of PPAR agonist treatments on hepatic expression of pro-inflammatory cytokines and pro-oxidant molecules. With regard to the cytokines, we focused on measuring IL-1β, IL-6, and TNF-α because these have been deemed the most relevant overall to the pathogenesis of alcoholic and non-alcoholic steatohepatitis [38,51]. Our findings revealed heterogeneity in responses related to chronic ethanol feeding, with or without PPAR agonist treatments. The most important observation was that cytokine activation in chronic ethanol-exposed livers was not suppressed by PPAR agonist treatments. In fact, this result is not surprising since the rats were continuously exposed to high levels of ethanol throughout the study and therefore the toxic insults were ongoing or sustained. However, despite ongoing injury and inflammation, treatment with the PPAR-δ or PPAR-γ agonist effectively restored hepatic function, thereby arguing against the concept that inflammation per se is the primary driving force in chronic alcoholic liver disease. Therefore, while cell injury, apoptosis, necrosis, inflammation, and pro-inflammatory cytokine activation are likely to initiate ALD, the cascade of hepatic degeneration is probably driven by dysregulated lipid metabolism, and organelle dysfunction, i.e. mitochondria and ER. This concept is supported by findings in a recent study showing that anti-inflammatory/anti-oxidant treatment of chronic ALD reduced inflammation and pro-inflammatory cytokine activation, but it did not restore hepatic insulin signaling or reduce hepatic expression of genes that mediate lipotoxicity [13].

Finally, we examined the effects of PPAR agonist treatments on indices of oxidative stress in experimental chronic ALD because of the known role of PPARs as regulators of proinflammatory cytokine responses [52,53]. As expected, hepatic 4-HNE and N-Tyr levels were both significantly elevated in chronic ethanol-fed rats. Treatment with the PPAR-δ agonist produced the best effects with respect to reducing 4-HNE and N-Tyr levels and rendering them either similar to or below those measured in control livers. The reductions in 4-HNE correspond with the reduced levels of steatosis (histology, Nile Red, EM) and restoration of mitochondrial structure (reflecting improved metabolism) correspond with PPAR-δ agonist-mediated reductions in lipid peroxidation (4-HNE), which itself could drive hepatocellular injury, turnover, and degeneration. On the other hand, despite structural improvements effectuated by the PPAR-γ agonist treatments, 4-HNE levels were significantly elevated, which may correspond to the persistence of hepatic steatosis.

In summary, this study demonstrates that chronic alcoholic liver disease can be rescued by treatment with PPAR agonists, similar to those used for diabetes mellitus and nonalcoholic steatohepatitis [28–32]. Corresponding with our previous results, we found that relatively low doses of PPAR agonists were effective for treating chronic ALD, despite continued high-level ethanol exposures. These results provide convincing evidence that liver structure and function can be restored by bolstering insulin/IGF sensitivity/responsiveness, which is needed for energy metabolism, gene expression and liver remodeling after injury. The three major histological and ultrastructural responses linked to PPAR agonist-mediated therapeutic effects on hepatic architecture are, reduced steatosis, reduced populations of mega-mitochondria, and restoration of the RER integrity. It is noteworthy that insulin resistance, steatosis, and mitochondrial dysfunction promote lipotoxicity with lipid peroxidation, ER stress, and oxidative stress, which together worsen insulin resistance, impair cell survival, and promote inflammation [19]. Future studies will determine the degrees to which specific inhibitors of ER stress and lipotoxicity restore hepatic structure and function in chronic alcohol-relative liver disease. The findings herein lend support to the concept that chronic alcoholic liver disease can be effectively treated with relatively low-dose PPAR-δ and/or PPAR-γ agonists.

## Figures and Tables

**Table 1:** Effects of PPAR Agonist Treatments on Liver Lipid Content in Chronic Alcohol-Induced Liver Disease.

Treatment	Control	Ethanol	t-statistic	P< Value
Vehicle	6.54 ± 0.59	11.21 ± 1.12	2.601	0.05
PPAR-α	11.27 ± 1.75	31.35 ± 3.06	12.60	0.0001
PPAR-δ	5.28 ± 0.53	7.35 ± 0.41	1.406	N.S.
PPAR-γ	9.47 ± 1.07	14.92 ± 1.10	4.017	0.001

Liver tissue homogenates were analyzed for lipid content using the Nile Red fluorometric assay. Values reflect mean ± S.E.M. fluorescence light units corrected for protein content in the sample. 2-way ANOVA tests demonstrated significant effects of ethanol (F=113.42; df=84; P<00001), PPAR agonist treatments (F=50.19, df=84; P<0.0001), and interactions between ethanol exposure and PPAR agonist treatments (F=30.56, df=84; P<0.0001). Bonferroni posttests demonstrated significant between group differences as indicated by the t-statistic and P-value in the Table.

**Table 2:** Effects of PPAR Agonist Treatments on Peri-sinusoidal Fibrosis in Chronic Alcohol-Induced Liver Disease.

Treatment	Control	Ethanol
Vehicle	Absent	Extensive
PPAR-α	Absent	Moderate
PPAR-δ	Absent	Extensive
PPAR-γ	Absent	Minimal

Formalin-fixed paraffin-embedded histological sections of liver from control or chronic ethanol fed adult rats that were treated with vehicle or a PPAR agonist, were stained with Sirius Red to detect peri-sinusoidal fibrosis. Peri-sinusoidal Sirius red staining was absent in control livers, irrespective of PPAR agonist treatment. In livers from Ethanol+Vehicle and Ethanol+PPAR-δ agonist treated rats, abundant peri-sinusoidal Sirius Red staining with a chicken-wire pattern was observed in hepatic Zones 2 and 3. Ethanol fed rats that were treated with a PPAR-α agonist had reduced peri-sinusoidal fibrosis relative to vehicle, and ethanol fed rats that were treated with the PPAR-γ agonist had minimal or no evidence of peri-sinusoidal fibrosis, similar to controls. (See Figure 4).
